# Supplementary material for: Automated next-generation profiling of genomic alterations in human cancers
Source: Nat Commun. 2022 May 20;13:2830. doi: 10.1038/s41467-022-30380-x (PMC9123004; doi:10.1038/s41467-022-30380-x)
Supplement: Supplementary file 1 — Supplementary Information [file 41467_2022_30380_MOESM1_ESM.pdf]

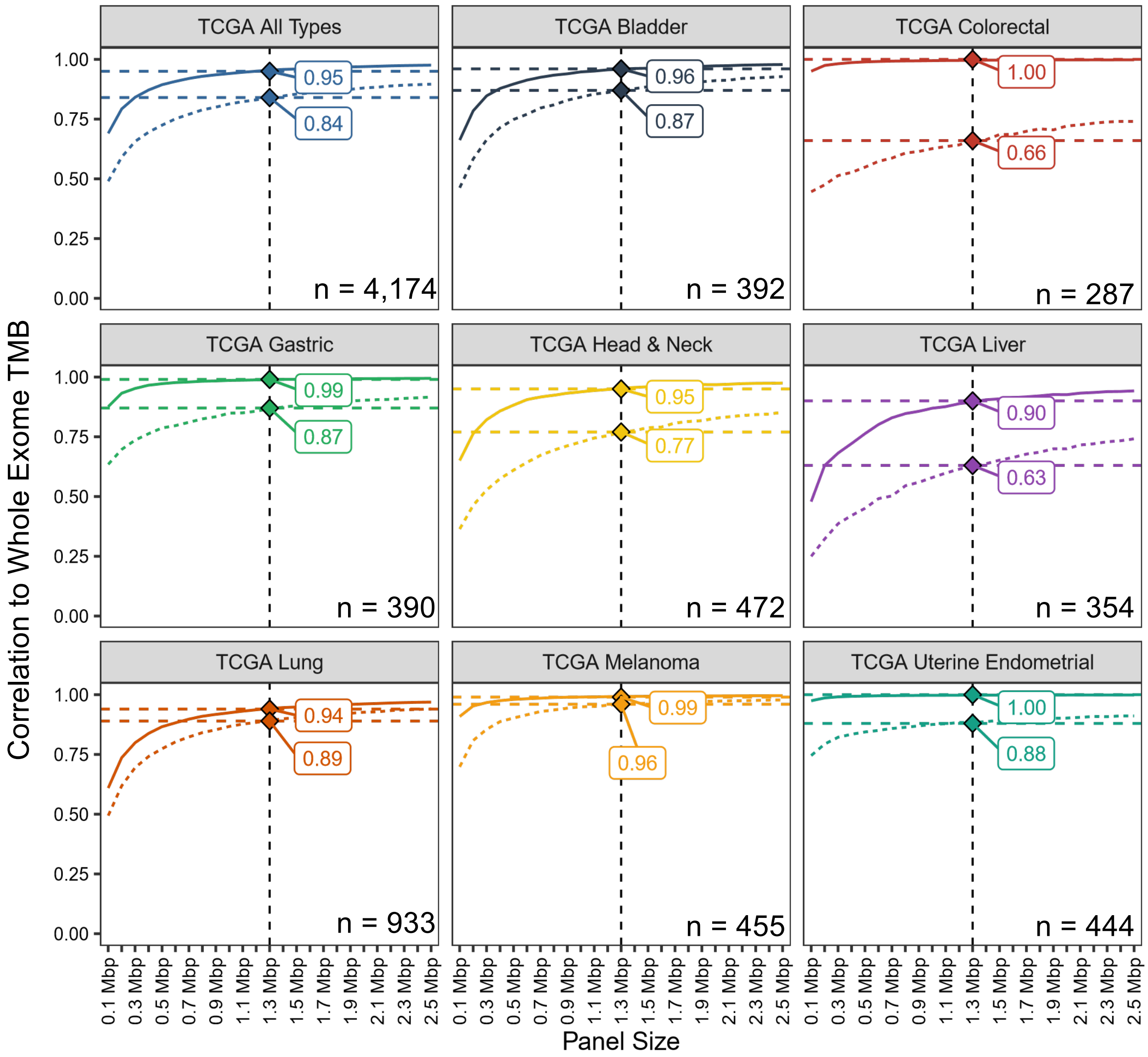

Supplementary Fig. 1. In silico tumor type comparison of targeted panel and whole exome TMB performance. The correlation of in silico predicted tumor mutation burden (TMB) for panels of different size (100 kilobases to 2.5 megabases, Mbp) to observed whole-exome TMB from The Cancer Genome Atlas (TCGA) samples of indicated tumor types, weighted according to relative frequency of Surveillance, Epidemiology, and End Results (SEER)-estimated new cases of metastatic cancer per year. Pearson correlation is represented by a solid line and Spearman correlation is represented by a dashed line. These analyses suggested that panels of >1 Mbp provide accurate TMB measurements. Source data are provided as a Source Data file.

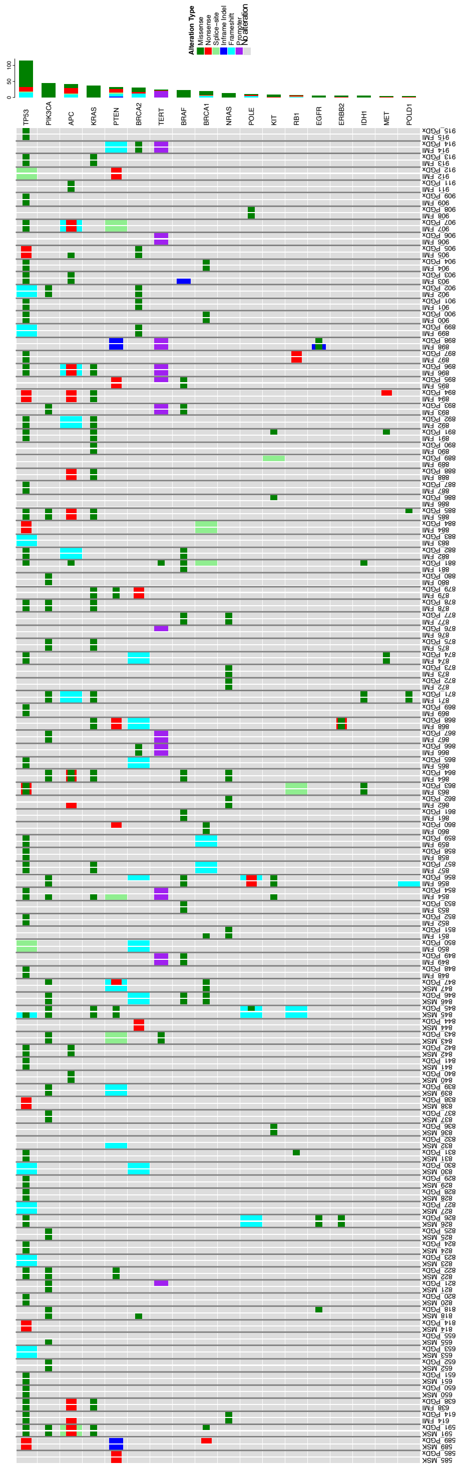

Supplementary Fig 2. Comparison of elio tissue complete to independent next generation sequencing results. A side-by-side comparison of detected sequence alterations between elio tissue complete (PGDx) and MSK-IMPACT (MSK) or FoundationOne (FMI) in select cancer driver genes. Corresponding samples are displayed next to each other, with the detected alterations for each gene colored as noted in the figure legend. Multiple alterations detected in one gene are denoted by overlapping colored rectangles. Source data are provided as a Source Data file.

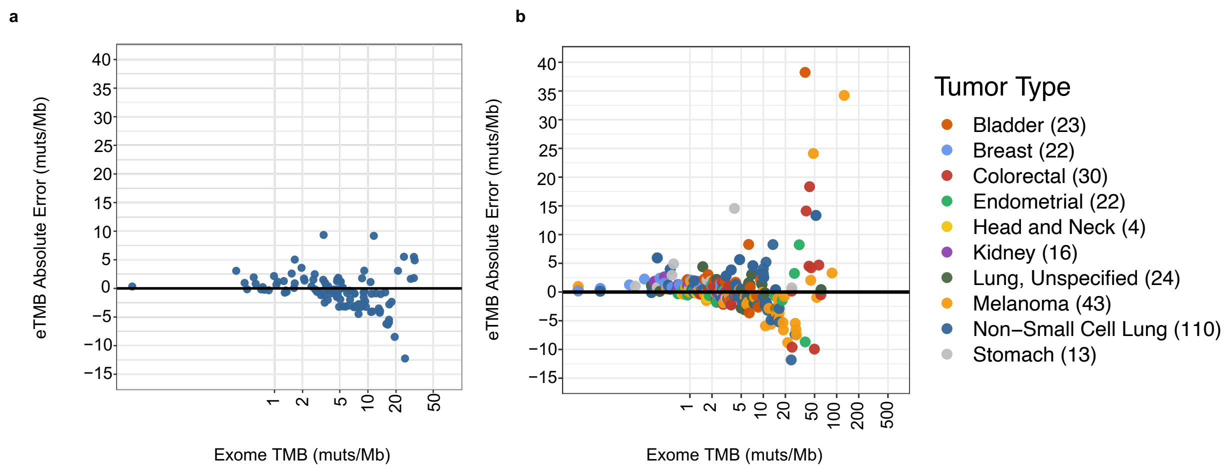

Supplementary Fig. 3. Absolute error observed in the analytical validation of the eTMB algorithm in non-small cell lung cancer and a pan-solid tumor cohort. a, After training the elio predicted exome tumor mutation burden (eTMB) algorithm using elio tissue complete (tumor-only) and whole exome sequencing (WES) (tumor and patient-matched normal) with a cohort of 106 formalin-fixed paraffin embedded (FFPE) and cell-line derived non-small cell lung cancer samples, we observed a mean absolute error from the WES tumor mutation burden (TMB) of 2.2 mutations/megabase (muts/Mb), exome equivalent and a median absolute error of 1.5 mutations/Mb, exome equivalent. **b,** When applied to a pan-solid tumor cohort (n = 307, number of each tumor type specified in parentheses), the eTMB had a mean absolute error of 3.4 mutations/Mb, exome equivalent and a median absolute error of 1.3 mutations/Mb, exome equivalent when compared to TMB obtained through WES. One sample with a WES TMB score of 602.5 mutations/Mb and an absolute error of 318.1 mutations/Mb, exome equivalent is omitted from the figure for display purposes. Source data are provided as a Source Data file.

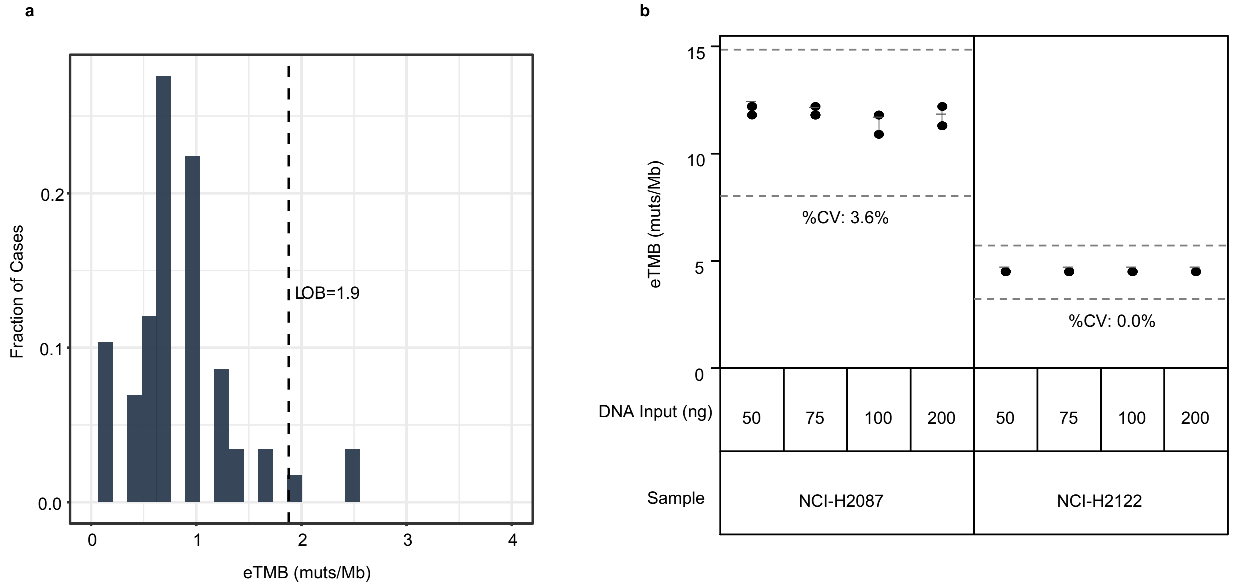

Supplementary Fig. 4. Limit of blank and effect of DNA input on the determination of eTMB. a, The limit of blank (elio predicted exome tumor mutation burden, eTMB, in noncancerous samples) was calculated to be 1.9 mutations/megabase (muts/Mb), exome equivalent, through identification of the 95th percentile of reported eTMB in a cohort of 58 formalin-fixed paraffin embedded (FFPE) samples derived from post-mortem noncancerous tissue. **b,** The variability of eTMB across a range of DNA input yields was determined through percent error from the mean eTMB (grey bar) observed at 100 nanograms (ng) of DNA input. Replicates at each DNA input had <30% deviation (represented by grey dashed lines) from the reference eTMB, with a median observed error of 2.61% and 0.00% for NCI-H2087 and NCI-H2122, respectively. Source data are provided as a Source Data file. LOB, Limit of Blank; CV, coefficient of variation.

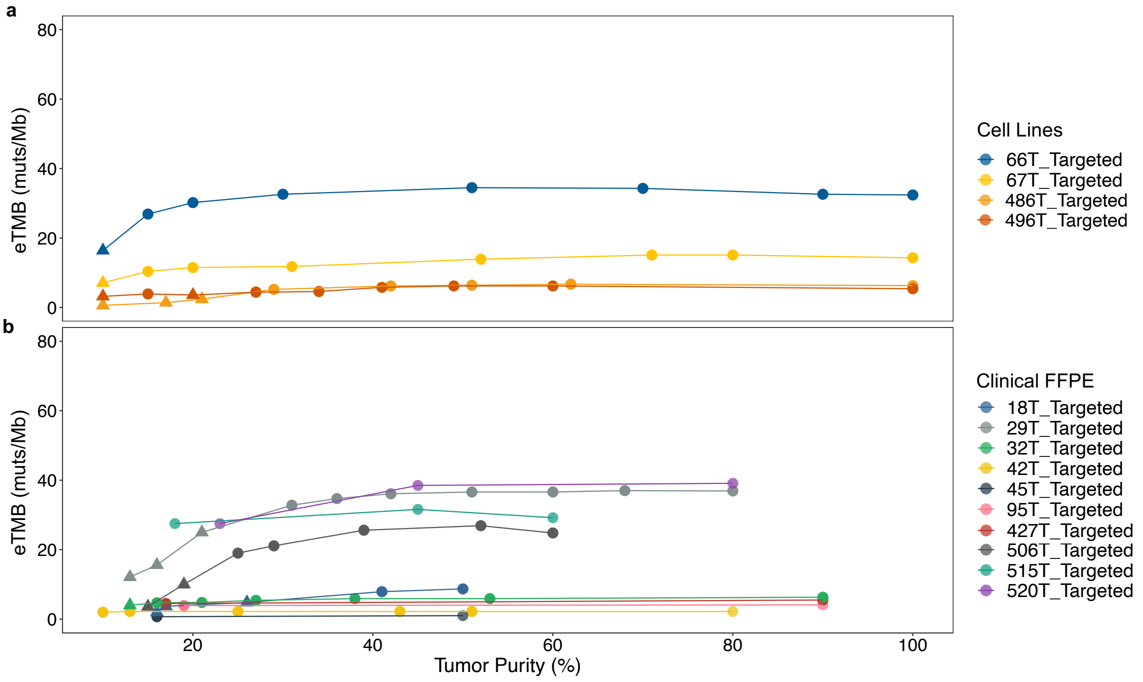

Supplementary Fig. 5. Effect of tumor purity on the determination of eTMB. Analytical sensitivity of the elio predicted exome tumor mutation burden (eTMB) across a range of tumor purities was measured in four tumor cell lines **a,** and 10 high purity formalin-fixed paraffin embedded (FFPE)-derived clinical tumor specimens **b**. The mean eTMB was taken across replicates at each observed tumor purity to determine eTMB at each dilution level. Triangles indicate the eTMB deviated >30% from the reference eTMB. Fewer than six of 14 cases deviated >30% from the reference eTMB at a tumor purity above 20%, demonstrating consistent analytical performance across a broad range of tumor purity values. Source data are provided as a Source Data file. muts/Mb, mutations per megabase.

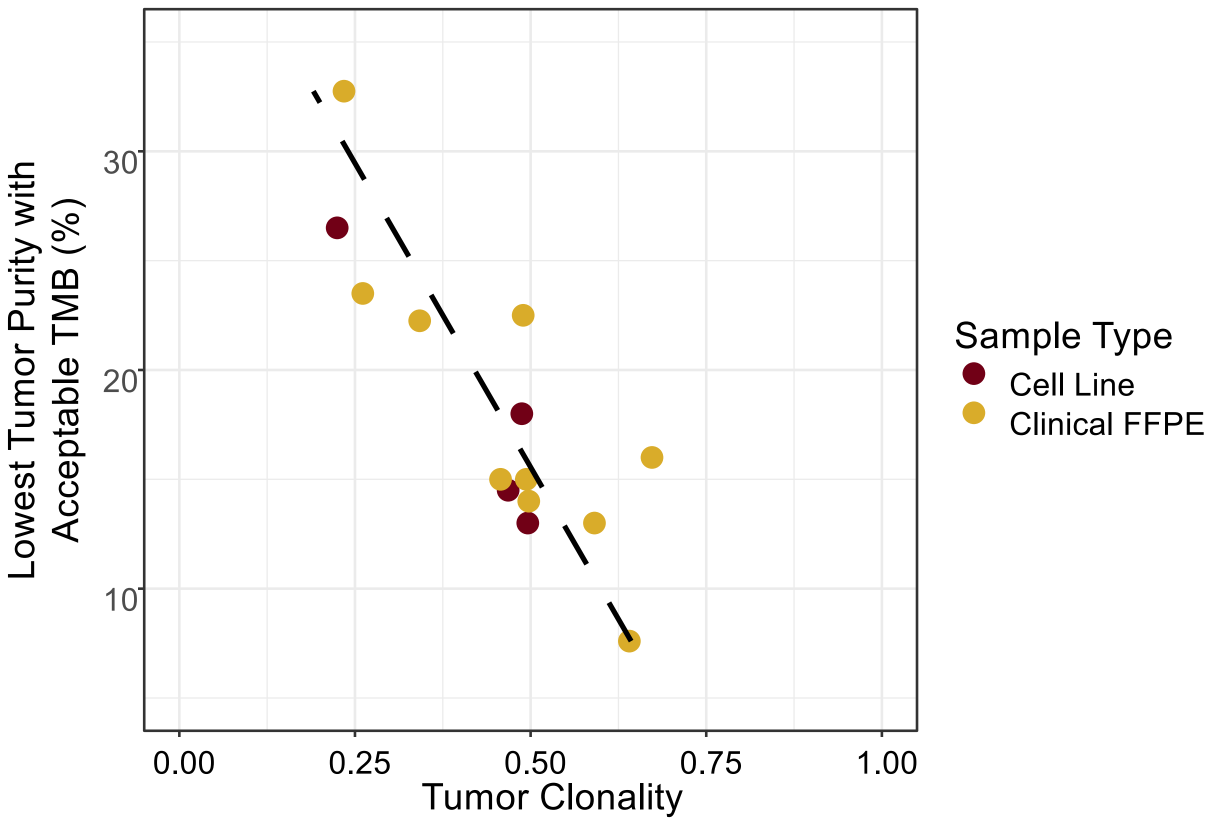

Supplementary Fig. 6. Effect of tumor heterogeneity on accurate eTMB estimates in samples with low tumor content. A set of four tumor cell lines and 10 formalin-fixed paraffin embedded (FFPE)-derived clinical tumor specimens were diluted with matched normal DNA to at least 5 dilution levels. The lowest observed tumor purity with an elio predicted exome tumor mutation burden (eTMB) deviation of <30% from the undiluted reference eTMB was determined (acceptable tumor mutation burden, TMB). Additionally, on the horizontal axis, the fraction of reads harboring a variant, corrected for tumor purity, as an estimate of tumor clonality is displayed. The tumor clonality was estimated by dividing the median sequence variant mutant allele fraction by the pathological tumor purity. We observed a negative correlation between the estimated tumor clonality and the lowest tumor purity with a deviation <30% (Pearson correlation = -0.840, p = 0.0002), suggesting TMB estimation may be affected by low tumor content (<35%) in heterogenous tumors. The Pearson correlation coefficient was calculated using a two-sided test, and no adjustments were made for multiple comparisons. Source data are provided as a Source Data file.

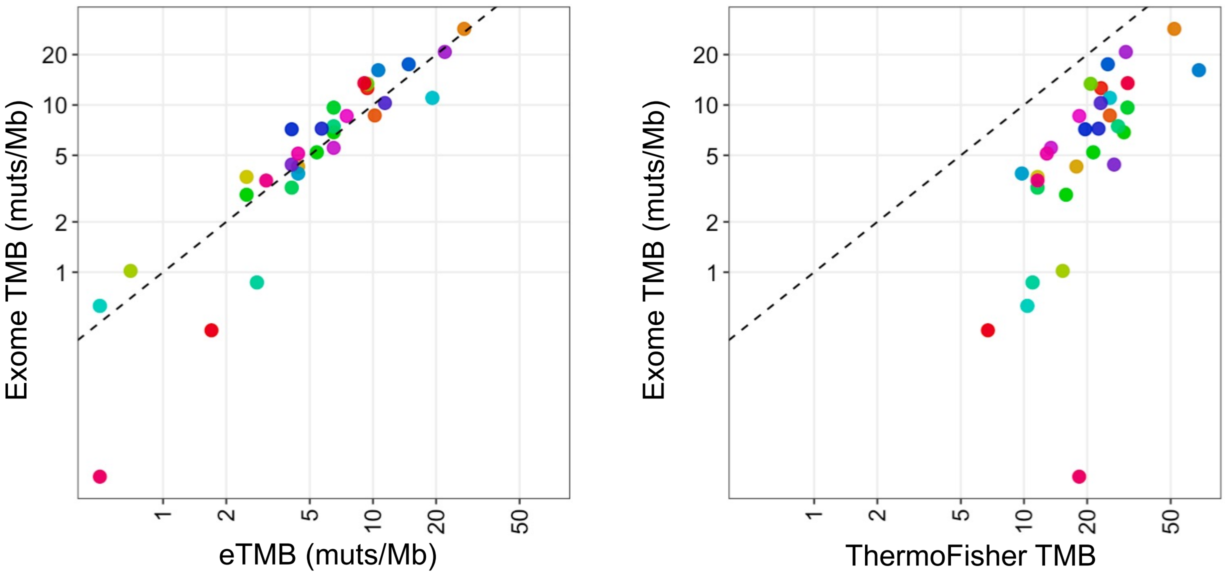

Supplementary Fig. 7. Comparison of TMB measurements by elio tissue complete or ThermoFisher Oncomine Tumor Mutation Load to Whole Exome Sequencing TMB. Thirty-one non-small cell lung cancer formalin-fixed paraffin embedded (FFPE) clinical tumor specimens were processed through the elio tissue complete test (left) and by the ThermoFisher Oncomine Tumor Mutation Load assay (right). Using a tumor mutation burden (TMB) estimation algorithm that was not trained on the analyzed samples, the elio predicted exome TMB (eTMB) showed higher accuracy to whole exome sequencing TMB as determined by Strelka2 than the ThermoFisher assay (Pearson correlation = 0.926 and 0.748, respectively). Source data are provided as a Source Data file. muts/Mb, mutations per megabase.

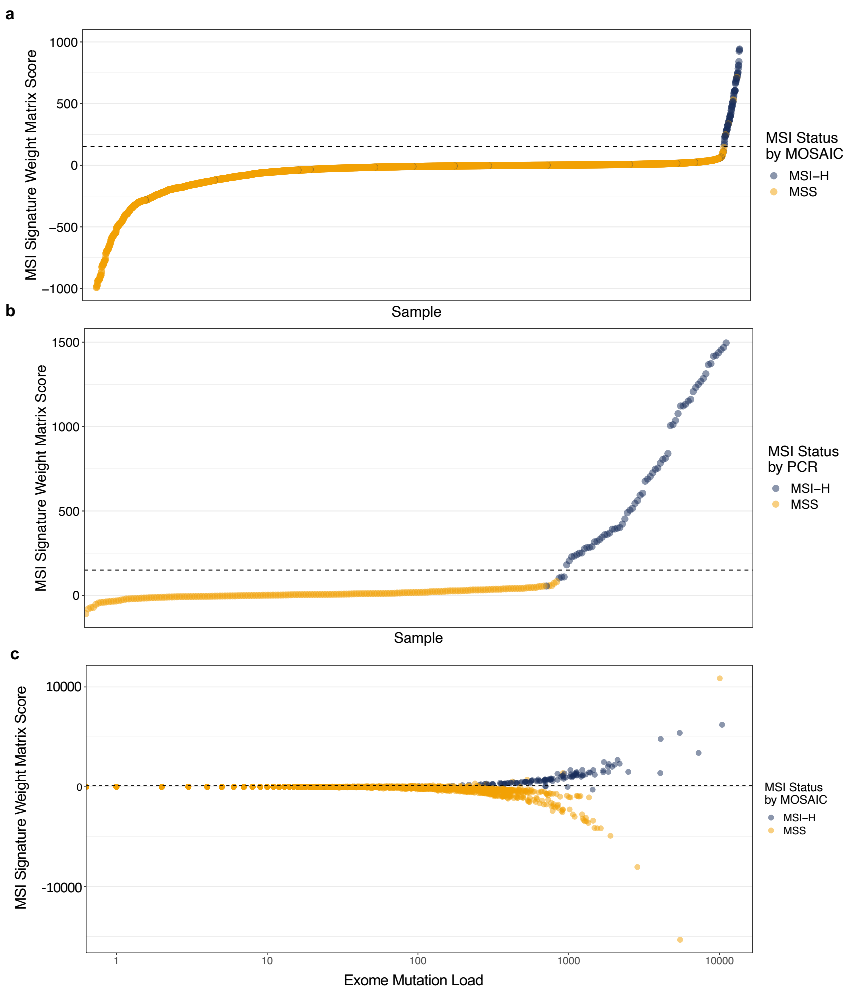

Supplementary Fig. 8. Feasibility analysis of the substitution scoring matrix for the detection of MSI. A position weight matrix (PWM) model to represent the mutation signatures associated with mismatch repair (MMR) deficiency was developed using >2,500 cancer exomes from The Cancer Genome Atlas (TCGA). An independent cohort of 2,847 TCGA cancer exomes with previously obtained microsatellite instability (MSI) results through the MOSAIC[28] algorithm were analyzed with the substitution signature weight matrix. A subset of these exomes (n = 264) additionally had been tested with a PCR multiplex assay. A sample with a substitution signature weight matrix score of ≥150 (dotted line) was classified as microsatellite instability – high (MSI-H). We observed a high concordance between the substitution signature weight matrix score and the MOSAIC classification **a,** and MSI status by the PCR-based method **b**. The substitution signature weight matrix score classified samples with 99.2% overall percent agreement (95% CI: 98.8%, 99.5%) to the MOSAIC classification and 98.5% overall percent agreement (95% CI: 95.9%, 99.5%) to the PCR-based classification. For display purposes, 46 samples with a score > 1,000 and 58 samples with a score < -1,000 were removed from the plot in **a** and 10 samples with a score > 1,500 were removed from the plot in **b**. **c,** A high tumor mutation burden does not necessarily lead to a high MSI signature PWM score, indicating the signature score reflects MMR deficiency and not other mutagenic processes. The exome mutation load of each TCGA sample was calculated by filtering the MC3 call set for passing, protein coding, non-silent somatic mutations with >10x normal coverage, >10% mutant allele fraction, and >4 mutant reads in the tumor. Source data are provided as a Source Data file. MSS, microsatellite stable.

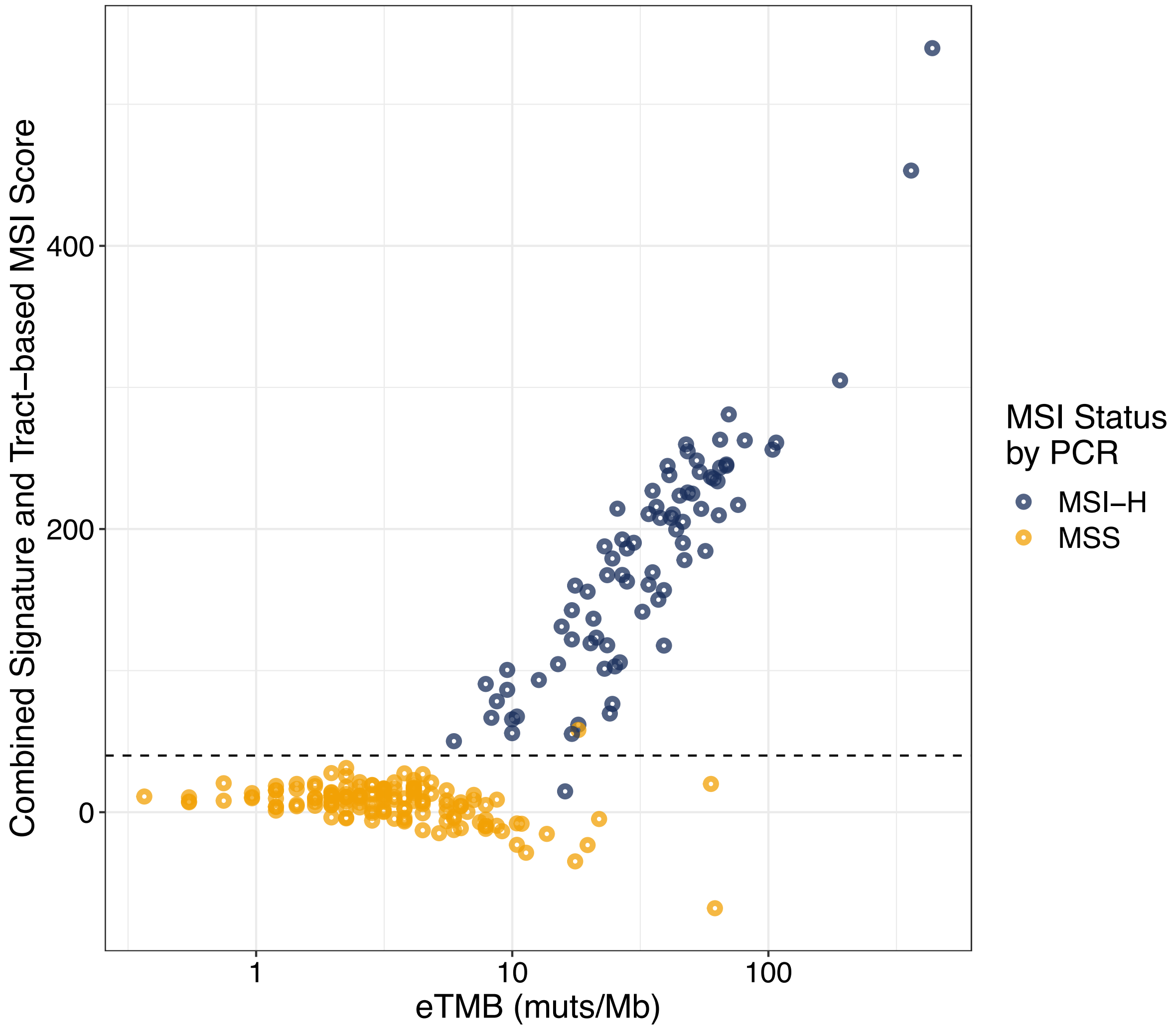

Supplementary Fig. 9. MSI algorithm is not affected by independent mutational processes. In a validation cohort of 223 formalin-fixed paraffin embedded (FFPE) tumors, high elio predicted exome tumor mutation burden (eTMB) scores were observed for microsatellite instability – high (MSI-H) patients as expected. A smaller cohort of high eTMB, microsatellite stable (MSS) patients were not classified as MSI-H by the microsatellite instability (MSI) algorithm, demonstrating that the signature score measures the mutational signatures of mismatch repair deficiency but not other DNA repair or mutagenic processes that may be inherent to a tumor. Source data are provided as a Source Data file. muts/Mb, mutations per megabase.

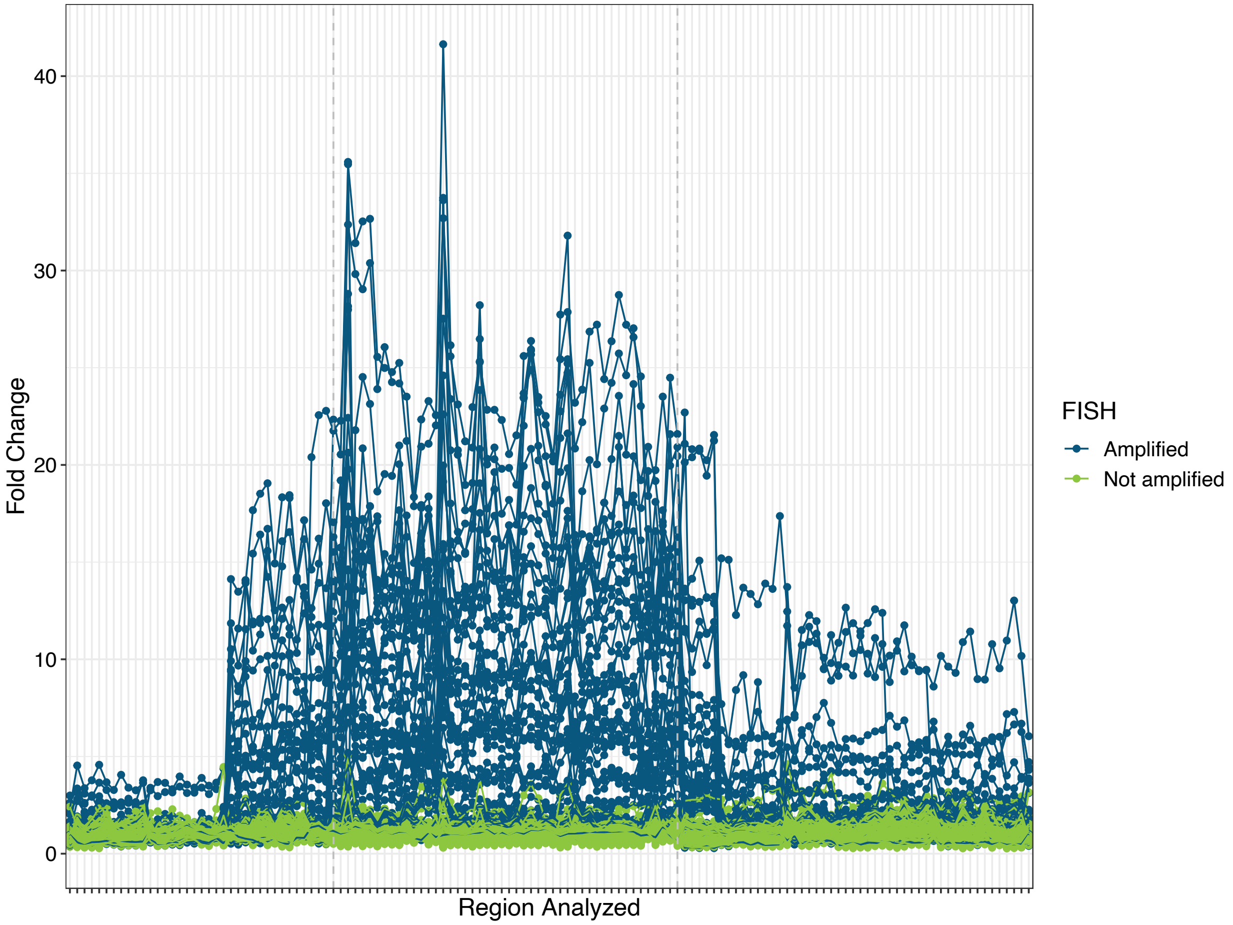

Supplementary Fig. 10. Concordance of *ERBB2* fold change measured by elio tissue complete with FISH. In a cohort of 120 formalin-fixed paraffin embedded (FFPE)-derived tumor specimens, high concordance to fluorescence in-situ hybridization (FISH)-derived results was observed. Each point represents the fold change for an individual region (*ERBB2* targeted gene regions lie within dashed lines), and points from the same sample are connected by a line. Lines are colored by the FISH results. The majority of discordant samples had fold change values close to the threshold for *ERBB2* amplification (average fold change of all regions >2.5). Source data are provided as a Source Data file.
