# Supplementary material for: Automated next-generation profiling of genomic alterations in human cancers
Source: Nat Commun. 2022 May 20;13:2830. doi: 10.1038/s41467-022-30380-x (PMC9123004; doi:10.1038/s41467-022-30380-x)
Supplement: Supplementary file 4 — Reporting Summary [file 41467_2022_30380_MOESM4_ESM.pdf]

nature portfolio

Corresponding author(s): Mark Sausen, PhD

Last updated by author(s): May 6, 2022

Reporting Summary

Nature Portfolio wishes to improve the reproducibility of the work that we publish. This form provides structure for consistency and transparency in reporting. For further information on Nature Portfolio policies, see our Editorial Policies and the Editorial Policy Checklist.

Statistics

For all statistical analyses, confirm that the following items are present in the figure legend, table legend, main text, or Methods section.

n/a	Confirmed	
☐	☒	The exact sample size (n) for each experimental group/condition, given as a discrete number and unit of measurement
☐	☒	A statement on whether measurements were taken from distinct samples or whether the same sample was measured repeatedly
☐	☒	The statistical test(s) used AND whether they are one- or two-sided<br>*Only common tests should be described solely by name; describe more complex techniques in the Methods section.*
☐	☒	A description of all covariates tested
☒	☐	A description of any assumptions or corrections, such as tests of normality and adjustment for multiple comparisons
☐	☒	A full description of the statistical parameters including central tendency (e.g. means) or other basic estimates (e.g. regression coefficient) AND variation (e.g. standard deviation) or associated estimates of uncertainty (e.g. confidence intervals)
☐	☒	For null hypothesis testing, the test statistic (e.g. F, t, r) with confidence intervals, effect sizes, degrees of freedom and P value noted<br>*Give P values as exact values whenever suitable.*
☒	☐	For Bayesian analysis, information on the choice of priors and Markov chain Monte Carlo settings
☒	☐	For hierarchical and complex designs, identification of the appropriate level for tests and full reporting of outcomes
☐	☒	Estimates of effect sizes (e.g. Cohen's d, Pearson's r), indicating how they were calculated

Our web collection on statistics for biologists contains articles on many of the points above.

Software and code

Policy information about availability of computer code

Data collection	Next-generation sequencing whole-exome sequencing reads were aligned to the hg19 human reference genome using ELAND (v1.8.2; www.illumina.com) and Novoalign (v3.2.7; www.novocraft.com) aligners. Next-generation sequencing targeted sequencing reads were aligned to the hg19 human reference genome using BWA-MEM [v0.7.15] and Bowtie2 [v2.3.1] aligners.
Data analysis	Sequence variants were identified using the VariantDx custom variant calling software (v9 and v10.4) and, for targeted analyses, assigned a confidence score by PGDx Cerebro (v20). Structural alterations were identified using the Digital Karyotyping and Personalized Analysis of Rearranged Ends methods adapted for this targeted panel as described in the manuscript. MSI status was determined using an adapted peak-finding approach together with a custom position weight matrix algorithm as described in the manuscript. Access to the previously published Cerebro machine learning framework for somatic sequence mutation discovery can be found in GitHub at http://github.com/PGDX/cerebro-paper along with the code to generate eTMB values at https://github.com/PGDX/tmb-paper.

For manuscripts utilizing custom algorithms or software that are central to the research but not yet described in published literature, software must be made available to editors and reviewers. We strongly encourage code deposition in a community repository (e.g. GitHub). See the Nature Portfolio guidelines for submitting code & software for further information.

Data

Policy information about availability of data

All manuscripts must include a data availability statement. This statement should provide the following information, where applicable:

- Accession codes, unique identifiers, or web links for publicly available datasets
- A description of any restrictions on data availability
- For clinical datasets or third party data, please ensure that the statement adheres to our policy

The de-identified raw sequence data and associated clinical variables data generated in this study have been deposited in the European Genome-Phenome Archive (EGA) database under accession code EGAS00001005556 as indicated in Supplementary Data 3 and 9. The de-identified raw sequence data and associated clinical variables data are available under restricted access where informed consent was provided for the release and publication of raw sequence data under an Institutional Review Board approved protocol, access can be obtained by contacting the PGDx elio tissue complete Validation Data Access Committee (https://ega-archive.org/dacs/EGAC00001002278) and will be made available for a minimum of one year. The raw sequence data where informed consent was not provided for release and publication are protected and are not available due to data privacy laws. In such cases, as well as those for which raw data is made available through EGA, the processed raw sequence data can be made available through a hosted PGDx elio tissue complete user interface, access can be obtained by contacting the PGDx elio tissue complete Validation Data Access Committee (https://ega-archive.org/dacs/EGAC00001002278). The public web resources used in this paper are listed here: the Surveillance, Epidemiology, and End Results (SEER) Program (https://seer.cancer.gov); dbSNP (v138, https://www.ncbi.nlm.nih.gov/projects/SNP/snp_summary.cgi?view+summary=view+summary&build_id=138); ExAC (v1, https://gnomad.broadinstitute.org/downloads#exac-variants); gnomAD (v2.0.2, https://gnomad.broadinstitute.org/downloads); COSMIC (v72, https://cancer.sanger.ac.uk/cosmic/download); and Synapse (syn7214402, https://www.synapse.org/#!Synapse:syn7214402/wiki/405297). The remaining data are available within the Supplementary Information and Source Data files. Source data are provided with this paper.

Field-specific reporting

Please select the one below that is the best fit for your research. If you are not sure, read the appropriate sections before making your selection.

[x] Life sciences   [ ] Behavioural & social sciences   [ ] Ecological, evolutionary & environmental sciences

For a reference copy of the document with all sections, see nature.com/documents/nr-reporting-summary-flat.pdf

Life sciences study design

All studies must disclose on these points even when the disclosure is negative.

Sample size	Sample sizes were selected per Clinical and Laboratory Standards Institute (CLSI) guidelines for analytical validation.
Data exclusions	Data not passing laboratory or bioinformatics quality control metrics are not included in the analytical accuracy, limit of blank, limit of detection, precision and reproducibility analyses.
Replication	After alteration-type specific novel algorithms were developed and custom software was adapted to the elio tissue complete targeted panel using a training dataset, validation of the analytical performance was assessed using an independent dataset. The analytical performance of the training and validation datasets is included for TMB (sequence mutations) and MSI analyses, while analytical performance for the validation set is listed for sequence and structural variant analyses.
Randomization	Samples were allocated to variant-type specific studies based on the alterations identified through pre-screening and/or orthogonal testing.
Blinding	Investigators were blinded to the orthogonal test results for the validation studies. Investigators were not blinded to the orthogonal test results for training studies, which were designed to enable optimization of method-specific algorithms utilizing these data.

Reporting for specific materials, systems and methods

We require information from authors about some types of materials, experimental systems and methods used in many studies. Here, indicate whether each material, system or method listed is relevant to your study. If you are not sure if a list item applies to your research, read the appropriate section before selecting a response.

Materials & experimental systems

n/a	Involved in the study	
[x]	[ ]	Antibodies
[ ]	[x]	Eukaryotic cell lines
[x]	[ ]	Palaeontology and archaeology
[x]	[ ]	Animals and other organisms
[ ]	[x]	Human research participants
[x]	[ ]	Clinical data
[x]	[ ]	Dual use research of concern

Methods

n/a	Involved in the study	
[x]	[ ]	ChIP-seq
[x]	[ ]	Flow cytometry
[x]	[ ]	MRI-based neuroimaging

Eukaryotic cell lines

Policy information about cell lines

Cell line source(s)	Human tumor and normal cells from previously characterized cell lines were obtained from ATCC (Manassas, VA, USA) (NCI-H1770 [CRL-5893]/NCI-BL1770[CRL-5960], NCI-H1672[CRL-5886]/NCI-BL1672[CRL-5959], NCI-H1395[CRL-5868]/NCI-BL1395 [CRL-5957], NCI-H1437[CRL-5872]/NCI-BL1437[CRL-5958], NCI-H2009[CRL-5911]/NCI-BL2009[CRL-5961], NCI-H2087 [CRL-5922]/NCI-BL2087[CRL-5965], NCI-H2122[CRL-5985]/NCI-BL2122[CRL-5967], NCI-H2126[CCL-256]/NCI-BL2126 [CCL-256.1D], NCI-H1184[CRL-5858]/NCI-BL1184[CRL-5949], NCI-H2171[CRL-5929]/NCI-BL2171[CRL-5969], NCI-H128 [HTB-120]/NCI-BL128[CRL-5947], HCC1008[CRL-2320], HCC1937[CRL-2336], NCI-H1975[CRL-5908], HCC1954[CRL-2338]/ HCC1954BL[CRL-2339], DLD-1[CCL-221], CHP-212[CRL-2273], NCI-H1650[CRL-5883], BT-474[HTB-20], and HCC1143BL [CRL-2362]) and Horizon Discovery (Waterbeach, UK) (HD753 and HD768).
Authentication	Cell lines were authenticated through STR genotyping.
Mycoplasma contamination	Cell lines were not tested for mycoplasma contamination.
Commonly misidentified lines (See ICLAC register)	No commonly misidentified lines were used during the conduct of this study.

Human research participants

Policy information about studies involving human research participants

Population characteristics	Tumor type for each subject was the only clinical covariate provided to the authors.
Recruitment	Patients were recruited by their respective institutions and were selected to enable the appropriate sample sizes included in each analytical study based on the presence, absence, or unknown status of specific biomarkers that may be reflected in each tumor specimen.
Ethics oversight	Institutional Review Board approval was provided by Duke Health Institutional Review Board (Pro00091621) and National Institutes of Health Clinical Center of the National Cancer Institute.

Note that full information on the approval of the study protocol must also be provided in the manuscript.
